# Common mental disorders and associated factors among Ethiopian prisoners: a systematic review and meta-analysis

**DOI:** 10.3389/fpsyt.2023.1214223

**Published:** 2023-07-07

**Authors:** Setegn Fentahun, Girmaw Medfu Takelle, Gidey Rtbey, Fantahun Andualem, Techilo Tinsae, Gebresilassie Tadesse, Mamaru Melkam

**Affiliations:** Department of Psychiatry, College of Medicine and Health Science, University of Gondar, Gondar, Ethiopia

**Keywords:** common mental disorders, prisoners, systematic review, meta-analysis, Ethiopia

## Abstract

**Background:**

Common mental disorders are a major public health concern in the world and negatively influence people’s psychological, physical, economic, and overall well-being. Since prisoners live with restricted freedom and lack regular social interaction like communication with family and friends, the prevalence of common mental disorders is frequently found to be higher among prisoners compared to the general population. Therefore, this systematic review and meta-analysis aimed to estimate the pooled prevalence of common mental disorders and their associated factors among prisoners in Ethiopia.

**Methods:**

The primary published studies were searched by using different databases like PubMed, Google Scholar, CINHAL, and African Journal Online. A total of eight primary articles that assessed the prevalence and associated factors of common mental disorders among Ethiopian prisoners were included in this systematic review and meta-analysis. The data were extracted using the data extraction format in a Microsoft Excel spreadsheet and exported to Stata version 14 for further analysis. The statistical heterogeneity was assessed by the *I*^2^ test. Due to the presence of heterogeneity among included studies, a random effect meta-analysis model was employed. Egger’s weighted regression test and funnel plots were used to check the presence of publication bias.

**Results:**

A total of 8 primary studies with 3,585 study participants were included in this systematic review and meta-analysis. The pooled prevalence of common mental disorders among Ethiopian prisoners was 62.29% with a 95% CI (50.94, 73.65). The subgroup analysis based on regional state revealed that the pooled prevalence of common mental disorders in Amhara, Oromia, and the other two regions (Addis Ababa and SNNPR) was 72.4, 50.23, and 54.25%, respectively. History of mental illness (AOR = 6.21, 95% CI: 3.27, 11.80), poor social support (AOR = 3.90, 95% CI: 1.45, 10.51), and traumatic life events (AOR = 3.63, 95% CI: 1.04, 12.73) were significantly associated with a common mental disorder in this review.

**Conclusion:**

In this systematic review and meta-analysis, the pooled prevalence of common mental disorders among prisoners was high. Therefore, improving the delivery of mental health services in correctional institutions has a major contribution to the reduction of the disorder, and incarcerated people require more attention and early intervention for common mental disorders.

## Introduction

Common mental disorders (CMDs) are groups of distress states that include anxiety, depression, and unexplained somatic symptoms, which are characterized by symptoms such as insomnia, fatigue, irritability, forgetfulness, difficulty concentrating, and other somatic complaints that may have a permanent impact on human life (1). They often co-occur with a fluctuating collection of symptoms that indicate emotional or mental abnormalities throughout the time that result in impairment of personal functioning (2, 3).

The worldwide burden of disease and disability is significantly impacted by common mental disorders, which are responsible for 14% of the overall disease burden (3, 4). They have been documented as being of the greatest public health concern, with an estimated 450 million individuals suffering from the problem worldwide (5). These common mental disorders (CMDs) have negative social and economic consequences in addition to a considerable functional disability (6, 7). By the year 2030, it is expected to be the main cause of illness burden. They have been well known as having frequent co-morbidities with other disorders (8). Of these frequent co-morbid mental diseases, depression and anxiety make up a higher percentage (8, 9). According to reports, women are more likely to develop CMDs than males, particularly in low- and middle-income countries (10, 11). Common mental disorders are more prevalent in low- and middle-income countries, with an estimated prevalence of 48.6% (12).

Although the prevalence of various mental disorders varies from one country to another countries, it is believed that 15 to 20% of all prisoners experience mental illness at any given time (13). Compared to the general population, serious psychiatric problems are 10 times more prevalent in correctional facilities, which is a higher prevalence than would be expected (14). Consistently, prisoners from numerous different countries are reported to have high rates of mental illness (15, 16). The overall magnitude of CMDs is high among prisoners, with ranges of 13 to 92.5% (17). Prison environments are characterized by stressful situations, overcrowding, violence, and isolation that expose inmates to loss of liberty, separation from family and friends, failure to maintain prior lifestyles and feelings of guilt and humiliation. As a result, many mental problems that may have already existed before imprisonment may get worse during incarceration (4, 18). People with mental illness are eight times more likely to experience sexual abuse in prison than prisoners without mental illnesses (19, 20).

Different studies in the world showed that common mental disorders are high among prisoners. Around 20% of female convicts and approximately 50% of male prisoners are estimated to have mental illnesses globally (21). European studies have revealed that 90% of inmates in the UK and 40% of prisoners in France experience mental health problems (22). In Hong Kong, 51.0 and 39.6% of lifetime histories of mental disorders and current mental disorders, respectively (23). According to studies conducted in various parts of the world, the prevalence of CMD is 65.3% in England, (23) 84.4% in India (24), 64% in the United States (25), 75% in Germany (26), and 73% in Canada (27). As previous studies conducted in Ethiopia, the prevalence of common mental disorders among prisoners varies largely and ranges from 37.9% (28) to 83.4% (29).

Epidemiological studies have identified several factors that are associated with common mental disorders among prisoners such as social exclusion, overcrowded prison settings, loss of autonomy, deprivation of decision-making, and loss of freedom and inadequate healthcare provisions in correctional service facilities (10, 30, 31). Low socioeconomic level, psychological illness, poor reproductive health, gender inequality, and physical disease are also risk factors for the common mental disorder (14, 30). Furthermore, pre-imprisonment factors like unemployment, lack of education, homelessness, a personal history of poverty, a history of drug use or abuse, and past mental illness significantly contribute to the development of common mental disorders among incarcerated people (32, 33). It is also well-recognized that understanding mental health problems depends on a person’s age (34). As a result, compared to older age groups, young prisoners have greater prevalence rates of mental health problems (34, 35).

If common mental disorders are not treated immediately, short-term and long-term physical, social, and vocational problems are inevitable (36, 37). The recurring nature of the problems, poor adherence, and treatment-seeking behavior all contribute to the long-term impact of common mental disorders (38). Common mental disorders have a negative influence on economic productivity, concomitant illnesses’ poor prognoses, disability, and health care costs (39). Mental health problems significantly affect the social, economic, educational, occupational, interpersonal, and physical condition of young prisoners (34, 35, 40).

Even though different primary studies were conducted in Ethiopia among prisoners to assess common mental disorders, their reports showed very discrepant and substantially inconsistent findings across the studies. Furthermore, to our knowledge, there is no previous systematic reviews and meta-analyses on the pooled prevalence and determinants of common mental disorders among prisoners in Ethiopia. Therefore, the main objective of this systematic review and meta-analysis was to identify the pooled prevalence of the common mental disorder among Ethiopian prisoners.

### Research questions

What is the pooled prevalence of common mental disorders among prisoners in Ethiopia?

What is the pooled effect size of associated factors with common mental disorders among prisoners in Ethiopia?

## Methods

### Protocol and registration

The protocol for the current systematic review and meta-analysis was registered in the international prospective register of systemic review (PROSPERO) (ID = CRD42023414004). We used an appropriate guideline for systematic reviews and meta-analyses reports, which is the Preferred Reporting Items for Systematic Reviews and Meta-Analyses (PRISMA-2020) checklists (41) (Additional file 1).

### Search strategy

Primary articles were searched by using PubMed/MEDLINE, Scopus, African Journal Online, PsycINFO, EMBASE, HINARI, Psychiatry Online, CINAL, Science Direct, and Cochrane Library. Google Scholar and Google were also used to search grey literature up to April 04/2023. A search strategy was developed based on the prevalence of common mental disorders, and associated factors for each database by using a combination of free texts and controlled vocabularies such as Mesh terms and keywords. The following key terms were used (Prevalence OR Magnitude OR Epidemiology OR Proportion) AND (Common mental disorders OR Common mental illness OR Mental illness OR Mental disorders OR Mental distress OR Psychological distress) AND (Associated factors OR Risk factors OR Determinants OR Predictors OR Correlate) AND (Prisoners OR Inmates OR Incarcerated OR Offenders OR Convicted OR Criminals) AND (Ethiopia).

### Eligibility criteria

This systematic review and meta-analysis included all observational studies like cross-sectional, case–control, and cohort study design on the prevalence of common mental disorders and associated factors among prisoners in Ethiopia published from 2013 to 2022. In this review, all articles with full text, and easily available, written in the English language, conducted in correctional institutions were included. However, articles without full articles, papers not written in the English language, case reports, case studies, conferences, and qualitative findings were excluded from this systematic review and meta-analysis. Moreover, studies that did not provide specific findings for the common mental disorder were also not included in this review.

### Data extraction

Two authors (GM and GT) independently extracted all the necessary data from the included primary articles using a standardized data extraction format organized in Microsoft Excel after the articles’ titles, abstracts, and complete texts were carefully reviewed. The final data extraction format contains the publication year, the first author’s name, the study design, the region where the study was conducted, the place of prison, assessment tools used to screen common mental disorders, sample size, and the prevalence of common mental disorders. As the second objective, factors associated with common mental disorders, including the 95% confidence interval and odds ratio were also extracted. Any discrepancies between the two authors that arise throughout the data extraction process were resolved by discussing with the third author (MM) and double extraction of the data that was inconsistent was done to attain common agreement.

### Outcome measurements

This systematic review and meta-analysis contain two main objectives. The first objective was to determine the pooled prevalence of common mental disorders among prisoners in Ethiopia. The second objective of this review was to identify the pooled effect size of associated factors with common mental disorders among Ethiopian prisoners. The pooled prevalence of common mental disorders was calculated using STATA version 14.0 and the pooled effect size of associated factors with common mental disorders was identified by using the odds ratio. The odds ratio was calculated from the reports of included primary studies by using two-by-two tables.

### Quality assessment

The quality of the primary studies included in this systematic review and meta-analysis was evaluated using the standard critical appraisal tool, which was done by two authors (TT and GR). The Joanna Briggs Institute (JBI) quality appraisal criteria were originally developed to evaluate the methodological quality of the prevalence of cross-sectional studies (42). This quality assessment instrument contains nine items with a range of scores from 0 to 9 (0–4 low, 5–7 moderate, and 8 and above high quality). Articles with quality scores of five and above were included in the present systematic review and meta-analysis. Any disagreements between authors in the quality assessment of the included articles were resolved by the third author (FA) to attain a common covenant.

### Data synthesis and analysis

The extracted data in the Microsoft Excel spreadsheet was exported to STATA 14.0 for further analysis. The results of this systematic review and meta-analysis are summarized and presented using texts, forest plots, and tables. The presence of statistical heterogeneity among included studies was assessed by using the *I*^2^ statistics test (43). Due to the existence of significant heterogeneity in this review, a random-effect meta-analysis model was conducted to estimate the pooled effect size of all involved studies at a 95% confidence interval. Subgroup analysis was conducted using publication year, assessment tools, and regional state to identify the source of heterogeneity. The effects of a single study on overall prevalence were checked using sensitivity analysis. Publication bias of the included studies was assessed by using both visual observation of the symmetry in the funnel plots and Egger weighted regression tests at a 5% significant level (44, 45). In Egger’s test, publication bias was considered to occur when the value of p was less than 0.05.

## Results

### Search results

**Figure 1:**
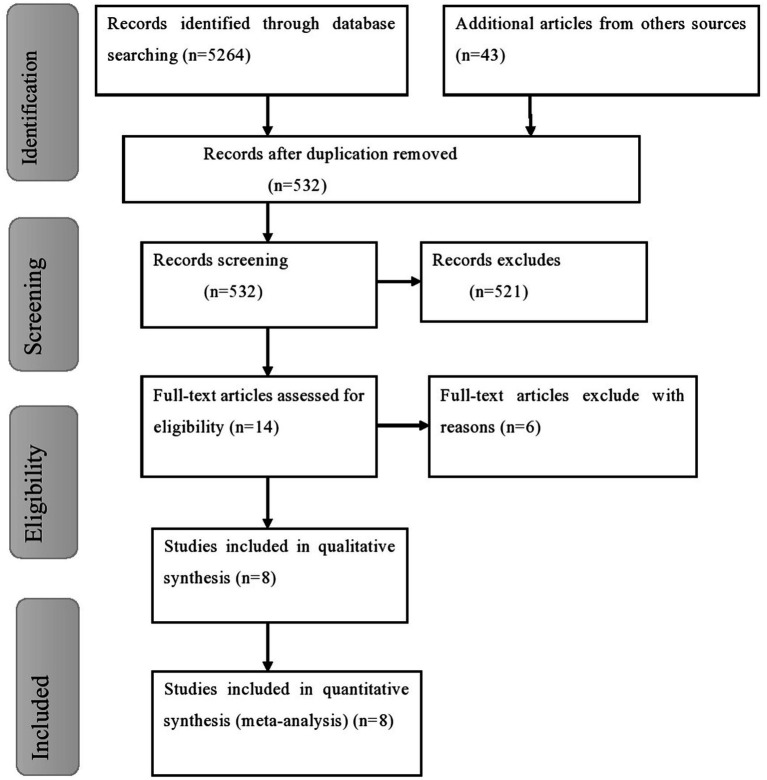
Flow chart of study selection for meta-analysis of common mental disorders among prisoners in Ethiopia.

Using various electronic search methods including PubMed, Google Scholar, CINHAL, and African Journal Online, a total of 5,307 studies were retrieved for this review. Among these studies, 4,775 were removed because of duplication. Furthermore, 521 studies were excluded after we looked at their titles and abstracts because they were irrelevant to our review, were not conducted in Ethiopia, had differences in study populations and settings, and their complete texts were unavailable. Another 14 full-text articles were reviewed for eligibility using the inclusion criteria, and 6 studies were excluded for other reasons. Finally, 8 studies were eligible and included in this systematic review and meta-analysis (Figure 1).

### Characteristics of included studies

In this systematic review and meta-analysis, a total of eight primary studies were included that were conducted on common mental disorders and their associated factors among prisoners in Ethiopia. The studies were carried out from October 2013 to February 2021 and published between 2013 and 2022. In the studies, four regional states (regions) of Ethiopia were included; four articles were conducted in Amhara (18, 29, 46, 47), two were in Oromia (28, 48), one was in SNNPR (49) and the other one study was conducted in Addis Ababa (50). A total of 3,585 participants were included, with sample sizes ranging from 302 (48) in Oromia to 700 (29) in the Amhara region. Concerning the assessment tools, two studies that were carried out at Addis Ababa and Bonga prisons were conducted by using the Kessler 10-item questionnaire, whereas the other six studies were done using a self-reported questionnaire (SRQ-20) to screen for common mental disorders. All primary studies included in this review were done using a cross-sectional study design. As reported from included studies, prisoners at Jimma prison, 37.9%, and Amhara prison, 83.4%, had the lowest and greatest prevalence of common mental disorders among Ethiopian prisoners, respectively (Table 1).

**Table 1:** Characteristics of studies included in this systematic review and meta-analysis on common mental disorder among prisoners in Ethiopia.

Authors	Publication year	Prisons	Assessment tools	Sample size	Prevalence of CMD in %
Solomon et al.	2019	Addis Ababa	SRQ-20	452	58.4
Yesuf et al.	2022	Amhara	SRQ-20	422	74.6
Ali et al.	2016	Debre Mrkos	SRQ-20	423	67.6
Adraro et al.	2019	Jimma	SRQ-20	302	62.7
Tegegne et al.	2022	Wollo	SRQ-20	413	63.6
Audu et al.	2013	Jimma	SRQ-20	546	37.9
Dachew et al.	2015	Amhara	K-10	700	83.4
Agegnew et al.	2019	Bonga	K-10	327	49.8

### The pooled prevalence of common mental disorders among prisoners in Ethiopia

**Figure 2:**
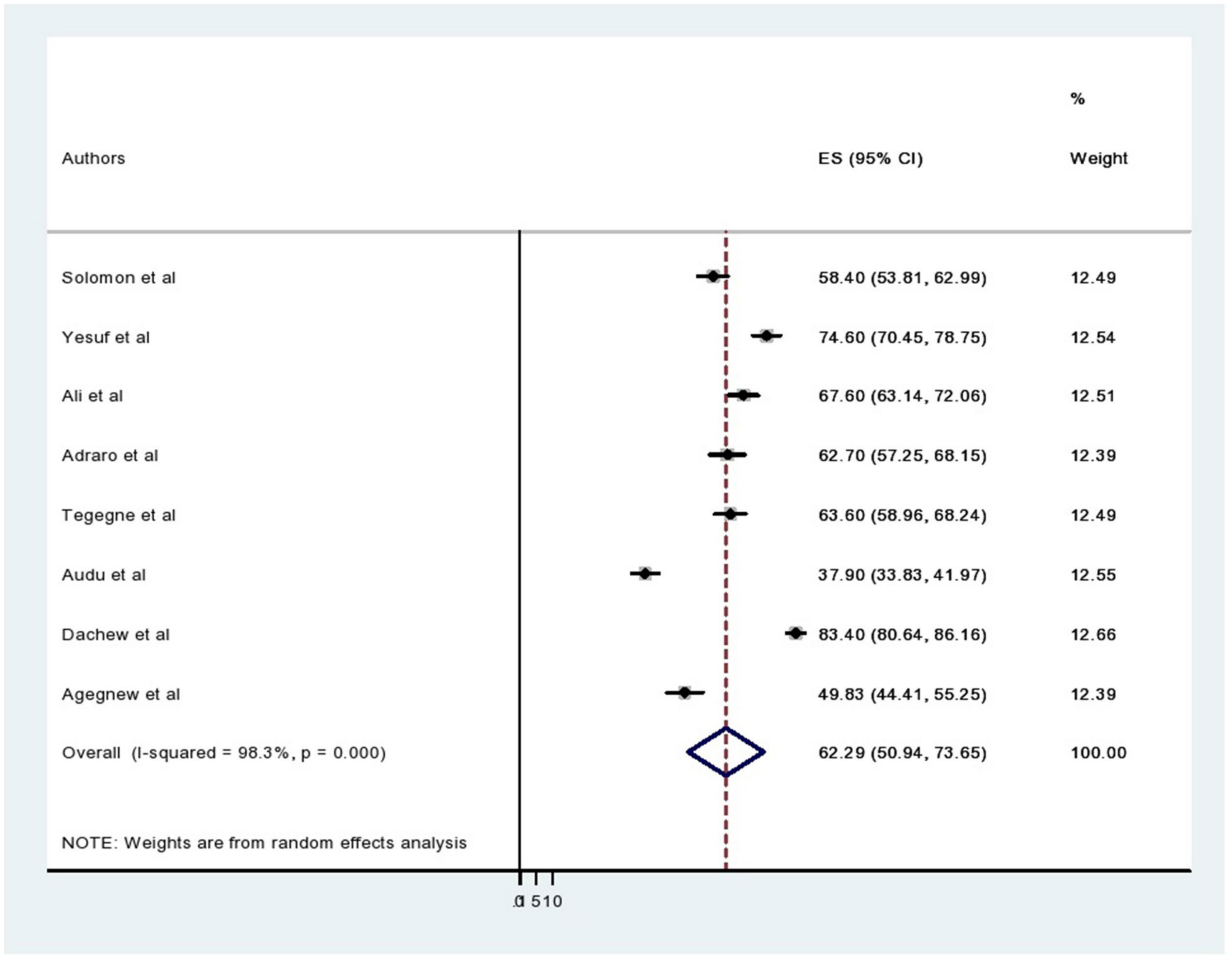
Forest plot showing the pooled prevalence of common mental disorders among prisoners in Ethiopia.

Eight studies were included in this review to determine the overall prevalence of common mental disorders among prisoners in Ethiopia. The pooled prevalence of common mental disorders among Ethiopian prisoners was found to be 62.29% with a 95% CI (50.94, 73.65) (Figure 2). In this systematic review and meta-analysis, the weighted prevalence of common mental disorders among regions was also done.

### Heterogeneity and publication bias

**Figure 3:**
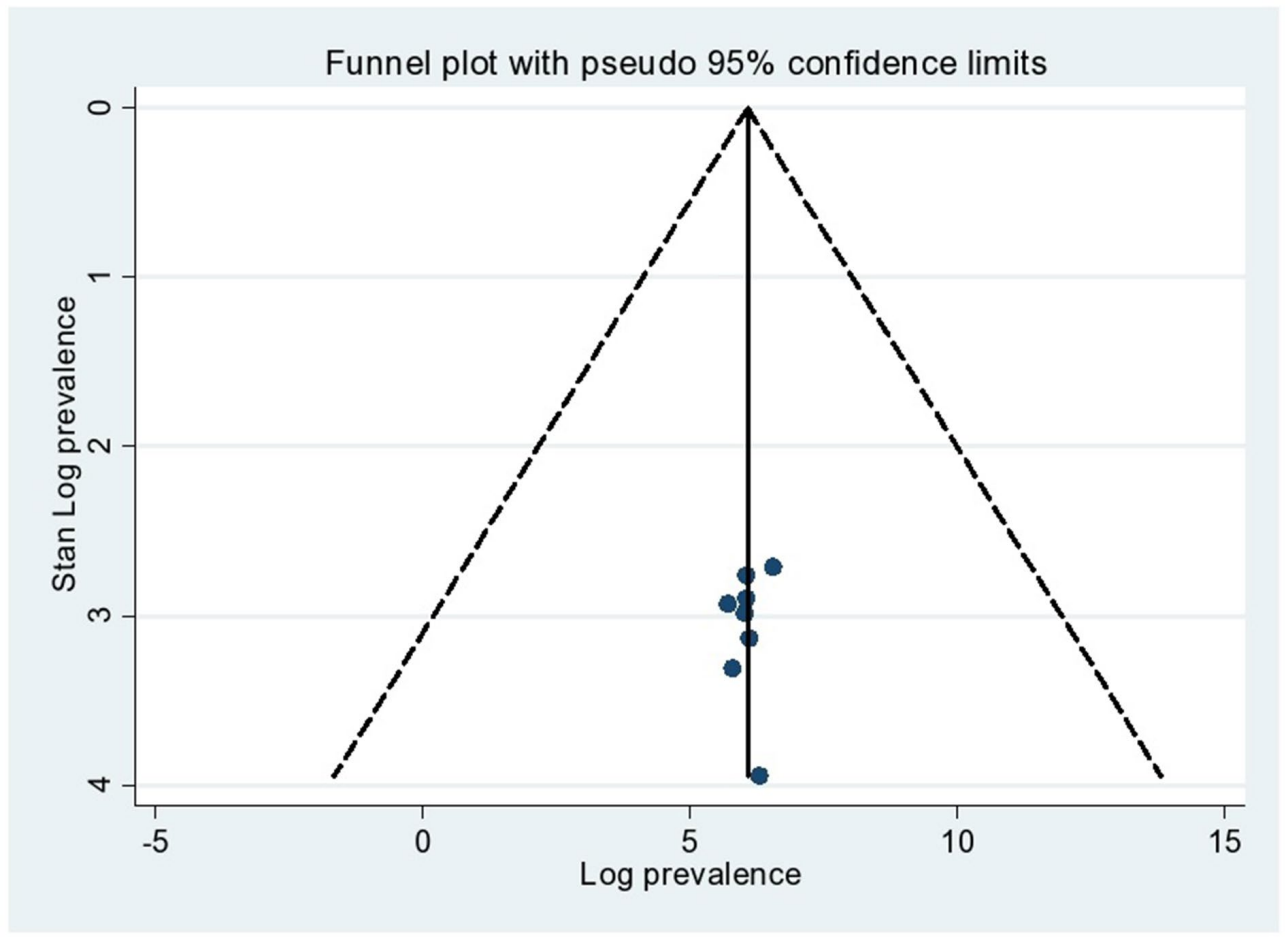
Funnel plot of common mental disorders among prisoners in Ethiopia.

The statistics test (*I*^2^) was conducted to assess the statistical heterogeneity, and there was a high level of heterogeneity in this study (*I*^2^ = 98.3%, value of *p* 0.001). Regarding publication bias, two techniques were used to check if there was a publication bias in the included studies. The first was checked by a funnel plot, which showed the symmetric distribution and revealed the absence of publication bias in the included articles (Figure 3). Furthermore, the absence of publication bias was also checked by the Eggers test as evidenced by *p* = 0.087 (Table 2).

**Table 2:** Eggre’s test of common mental disorder among prisoners in Ethiopia.

Std_Eff	Coef.	Std. Err.	*t*	*P* > *t*	[95% Conf. Interval]
Slope	0.6535	0.39511	5.08	0.002	0.30153 0.0054
Bias	0.58008	0.08589	0.04	0.087	0.25936 0.099207

### Subgroup analysis

Because the pooled prevalence of common mental disorders was affected by heterogeneity, subgroup analysis based on study region, publication year, and assessment tools used to measure common mental disorders was conducted. The subgroup analysis showed that the pooled prevalence of the common mental disorder among prisoners was highest in the Amhara region 72.41% (95% CI: 63.05, 81.77), followed by two regional studies (Addis Ababa and SNNPR) 54.25% (95% CI: 45.85, 62.64), and Oromia 50.23% (95% CI: 25.93, 74.53). The pooled prevalence of the common mental disorder among articles conducted by using SRQ-20 60.12% (95% CI: 49.03, 71.21) was lower than the pooled prevalence of the common mental disorder in articles that were measured by K-10 66.70% (95% CI: 33.8, 99.60). Furthermore, the pooled prevalence of common mental disorders was greater in the studies carried out after 2018 with a prevalence of 62.99% (95% CI: 35.49, 90.49) than the pooled prevalence of common mental disorders that were conducted before 2018 with the prevalence of 61.09% (95% CI: 52.39, 69.80; Table 3).

**Table 3:** Subgroup analysis of common mental disorder and associated factors among prisoners in Ethiopia.

Variables	Subgroup	Number of studies	Prevalence (95% CI)	*I*^2^ (%)	*p* value
Region	Amhara	4	72.41 (63.05, 81.77)	95.7	0.000
	Oromia	2	50.23(25.93, 74.53)	98.0	0.000
	Others	2	54.25 (45.85, 62.64)	82.3	0.018
Publication year	<2018	3	61.09 (52.39, 69.80)	93.9	0.000
	>2018	5	62.99 (35.49, 90.49)	99.4	0.000
Assessment tools	SRQ-20	6	60.12 (49.03, 71.21)	97.2	0.000
	K-10	2	66.70 (33.8, 99.60)	99.1	0.000

Others = Addis Ababa and SNNPR.

### Sensitivity analysis

In this systematic review and meta-analysis, the sensitivity analysis was carried out to examine the heterogeneity of those studies by systematically excluding one author or one study, to determine the impact of each study’s findings on the pooled prevalence of common mental disorders. As the results show, all of the values fall within the expected 95% CI, providing that the exclusion of a single study did not significantly alter the prevalence of this review (Table 4).

**Table 4:** Sensitivity analysis of common mental disorders among prisoners in Ethiopia.

Authors	Estimate 95% CI	Heterogeneity	
		*I* ^2^	*P* value
Solomon et al.	62.84 (50.03, 75.56)	98.5	≤0.001
Yesuf et al.	60.53 (47.65, 73.4)	98.4	≤0.001
Ali et al.	61.53 (48.53, 74.56)	98.5	≤0.001
Adraro et al.	62.23 (49.52, 74.94)	98.5	≤0.001
Tegegne et al.	62.10 (49.15, 75.06)	98.5	≤0.001
Audu et al.	65.84 (56.75, 74.93)	96.8	≤0.001
Dachew et al.	59.24 (49.46, 69.01)	96.8	≤0.001
Agegnew et al.	64.05 (51.92, 76.20)	98.3	≤0.001

### Factors associated with common mental disorders among prisoners

**Figure 4:**
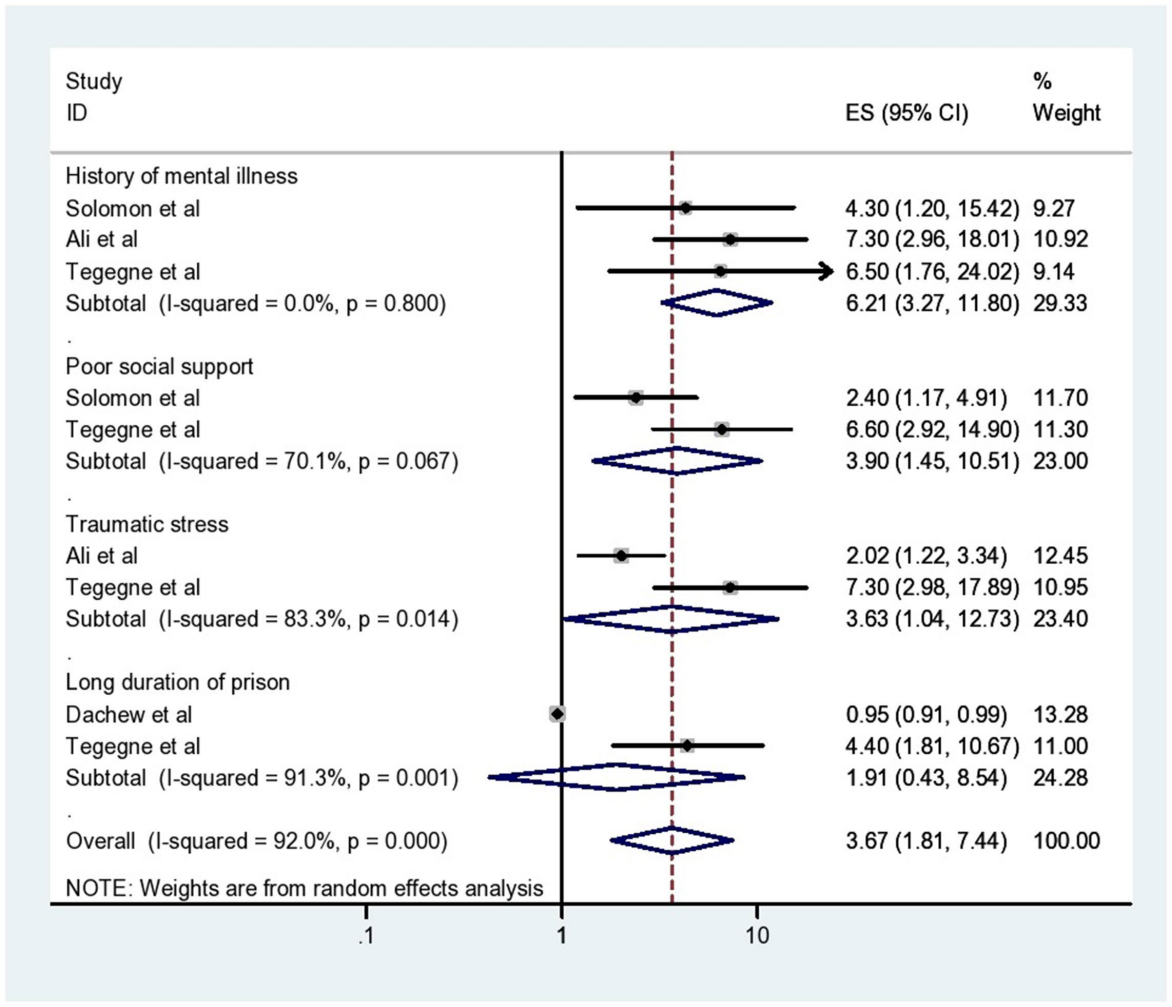
The forest plot showing different associated factors of common mental disorders among prisoners in Ethiopia.

From the included primary studies, there are different factors associated with common mental disorders among prisoners in this systematic review and meta-analysis in Ethiopia. Having a history of mental illness, poor social support, and traumatic life event were associated with common mental disorders among Ethiopian prisoners. History of mental illness was significantly associated with common mental disorders among the three primary articles included in this systematic review and meta-analysis. The weighted odds ratio revealed that having a history of mental illness was 6.21 times more likely to have a common mental disorder compared to participants without a previous history of mental illness (AOR = 6.21, 95% CI: 3.27, 11.80). In this systematic review and meta-analysis, those respondents who have poor social support were 3.9 times more vulnerable to common mental disorders than participants who have strong social support (AOR = 3.90, 95% CI: 1.45, 10.51). Traumatic life events were reported as an associated factor with common mental disorders between two primary articles among Ethiopian prisoners. Traumatic life events were 3.63 times more likely to develop a common mental disorder compared to their counterparts (AOR = 3.63, 95% CI: 1.04, 12.73) (Figure 4).

## Discussion

In this systematic review and meta-analysis, 3,585 prisoners were included. This study was conducted to estimate the pooled prevalence and associated factors of common mental disorders among Ethiopian prisoners. It is also the first systematic review and meta-analysis in Ethiopia among prisoners. The pooled prevalence of common mental disorders among included studies of Ethiopian prisoners was 62.29% with a 95% CI (50.94, 73.65). This finding is in line with different studies that were carried out in Kenya 63.2% (51), Zambia 63.10% (19), New Zealand 63.6% (52), Australia 63% (53), England 65.3% (54), and the United States 64% (25). The finding of this review is also consistent with the systematic review and meta-analysis were done in Africa among youth detained people with a pooled prevalence of 61% (55).

The Ethiopian pooled prevalence of common mental disorder was lower than other studies that were conducted in different countries like Nigeria 87.8% (56), Kenya 84% (57), Uganda 86% (4), Spain 84.4% (24), Germany75% (26), Canada 73% (27) and Brazil 68.9% (58). The possible reason for the discrepancy might be the difference in assessment tools. For example, the study conducted in Nigeria was measured by using General Health Questionnaire (GHQ-30), and the other study in Uganda was screened by using Mini International Neuropsychiatric Interview (MINI). The other difference might be the variation in sampled study participants. For instance, in Kenya, Brazil, and Germany, the studies were conducted among only female prisoners, who had a higher chance of vulnerability to common mental disorder (10).

The finding of the current systematic review and meta-analysis was higher than the results of other studies done in, Zambia 29.2% (59), Iran 43.4% (60), Cameron 34.8%, (61) United States 48% (62), and New South Wales, Australia 42.7% (63). The observed variation might be due to the fact that most other studies conducted in different countries had a single finding, whereas this study had a pooled prevalence from different studies. The additional discrepancy might be related to differences in the accessibility and delivery of mental health services in correctional settings, and variations in prison environments, such as work inside prison (4, 14). It could be also due to the difference in sample size and variation in socio-cultural among sampled study participants.

In addition to the pooled prevalence, in this review, determinants of common mental disorders were also determined among prisoners. The pooled effect of associated factors revealed that a history of mental illness, poor social support, and traumatic life events were significantly associated with common mental disorders among prisoners.

Accordingly, a history of mental illness was a significant predictor of common mental disorders among prisoners. This finding was supported by other studies conducted in Egypt (64). This might be due to the fact that Fear of social stigma and prejudice in prison, which inmates are preoccupied with and are likely to cause them distress, guilt about their illnesses, a lack of social support, and other stresses are all major sources of stress, and these may increase the likelihood of prisoners developing a common mental disorder (31). Another possible reason that may contribute to common mental disorder is that the stress of being incarcerated may make prisoners’ mental illnesses worse, and the impact of incarceration is so severe that it can lead to mental illness in those who are already vulnerable (14).

This systematic review and meta-analysis showed that prisoners with poor social support were more likely to develop a common mental disorder than prisoners who had good social support. This finding was consistent with other studies done in Netherlands (65), Iran (60), and Egypt (64). This could be because, a significant source of stress for those who are imprisoned includes having fewer close friends both inside and outside of prison, feeling less connected to family, and having fewer outside interactions through letters, phone calls, and visits, which contribute to the occurrence of prisoners common mental disorder (34). The other possible reason is also related, in addition to being the cause of mental illness, poor social support also affects the treatment outcomes of the disorder. Moreover, through positive assessment of the problems and a decrease in negative emotional responses, social support can improve the help-seeking and coping methods of prisoners (40).

This review also revealed that participants with traumatic life events were at higher risk of developing a common mental disorder than those without any traumatic life events. This finding was in line with the study conducted in the United States of America (66). This might be due to the fact that mental illness may manifest during imprisonment as a result of prison conditions such as social isolation, loss of connection with family and friends, restricted autonomy, lack of freedom, deprivation, and traumatic experiences (15, 26). The other additional explanation for this association is that the likelihood of developing a common mental disorder increases when a person experiences more stressful life events (20).

### Limitations of the study

Even though this systematic review and meta-analysis provides numerous advantages, the combined effect of common mental disorder among prisoners has the following limitation: all primary studies included in this review were conducted using a cross-sectional study design, which does not establish a real cause and effect relationship but only shows a temporal relationship. The other limitations of this review were that there was heterogeneity among included primary articles, and this systematic review and meta-analysis were done by including a small number of studies.

## Conclusion and recommendation

This systematic review and meta-analysis revealed that the pooled prevalence of common mental disorders among prisoners in Ethiopia was high. According to the findings of the subgroup analysis that was conducted using publication year, assessment tool, and regional state, there is a difference in the pooled prevalence of common mental disorders among prisoners. A history of mental illness, poor social support, and traumatic life events were recognized as significantly associated factors for the presence of common mental disorders. This indicated that prison administrators, the Ministry of Health, and policymakers should work collaboratively to increase and strengthen mental health facilities in correctional institutions, including early screening, detection, and interventions for mental health problems in prison. Moreover, it is necessary to provide some type of work inside prisons that may increase the unity among prisoners.

## Data availability statement

The original contributions presented in the study are included in the article/Supplementary material, further inquiries can be directed to the corresponding author.

## Author contributions

SF conceptualized the study and was involved in the design, searching strategy, extraction of data, analysis and review of the article, interpretation, report, and manuscript writing. GMT, GT, and MM were involved in the data extraction. TT, GR, and FA made substantial contributions to the quality assessment of the included studies and the drafting of the manuscript. All authors contributed to the article and approved the submitted version.

## Conflict of interest

The authors declare that the research was conducted in the absence of any commercial or financial relationships that could be construed as a potential conflict of interest.

## Publisher’s note

All claims expressed in this article are solely those of the authors and do not necessarily represent those of their affiliated organizations, or those of the publisher, the editors and the reviewers. Any product that may be evaluated in this article, or claim that may be made by its manufacturer, is not guaranteed or endorsed by the publisher.

## Abbreviations

AOR: Adjusted Odd Ratio
CI: Confidence Interval
CMD: Common Mental Disorder
GHQ-30: General Health Questionnaire
MINI: Mini International Neuropsychiatric Interview
SNNPR: South Nation Nationality and Peoples
SRQ-20: Self-Reported Questionnaire
USA: United States of America
