# Supplementary material for: Common mental disorders and associated factors among Ethiopian prisoners: a systematic review and meta-analysis
Source: Front Psychiatry. 2023 Jul 7;14:1214223. doi: 10.3389/fpsyt.2023.1214223 (PMC10360204; doi:10.3389/fpsyt.2023.1214223)
Supplement: Supplementary file 2 [file Table_2.docx]

Table S2**:** Quality assessment of common mental disorder and its associated factors among prisoners in Ethiopia included studies in this meta-analysis and systematic review.

| Authors | Q1 | Q2 | Q3 | Q4 | Q5 | Q6 | Q7 | Q8 | Q9 | Total score (9%) |
| --- | --- | --- | --- | --- | --- | --- | --- | --- | --- | --- |
| Solomon et al, 2019 | Y | Y | Y | Y | Y | Y | Y | Y | Y | 9 |
| Yesuf et al, 2022 | Y | Y | Y | Y | Y | Y | Y | Y | NR | 8 |
| Ali et al, 2016 | Y | Y | Y | Y | Y | Y | Y | Y | Y | 9 |
| Adraro et al, 2019 | Y | Y | NA | Y | Y | Y | Y | Y | Y | 8 |
| Tegegne et al, 2022 | Y | Y | Y | Y | Y | Y | Y | NA | Y | 8 |
| Audu et al, 2013 | Y | NA | Y | Y | Y | Y | Y | Y | NR | 7 |
| Dachew et al, 2015 | Y | Y | Y | Y | Y | Y | Y | Y | NA | 8 |
| Agegnew et al, 2019 | Y | Y | NA | Y | Y | NA | Y | Y | Y | 7 |

**Key:** **Y**= Yes; **NR**= Not reported, **NA**=Not appropriate

**Question codes:**

1. Was the sample frame appropriate to address the target population?

2. Were study participants sampled in an appropriate way?

3. Was the sample size adequate?

4. Were the study subjects and the setting described in detail?

5. Was the data analysis conducted with sufficient coverage of the identified sample?

6. Were valid methods used for the identification of the condition?

7. Was the condition measured in a standard, reliable way for all participants?

8. Was there appropriate statistical analysis?

9. was the response rate adequate, and if not, was the low response rate managed appropriately?
